# Semi-automated contact tracing and management of contact precautions during the COVID-19 pandemic within a tertiary hospital

**DOI:** 10.1016/j.infpip.2022.100266

**Published:** 2022-12-23

**Authors:** Lukas Bechmann, Gernot Geginat

**Affiliations:** Department of Medical Microbiology and Infection Control, Otto-von-Guericke University Magdeburg, Germany

**Keywords:** Covid-19, Contact tracing, Hospital, Tool, Transmission, Risk factor

## Abstract

**Background:**

Evaluation of a spreadsheet-based COVID-19 contact-tracing tool (CTT) and determination of risk factors for SARS-CoV-2 transmission among hospital staff members.

**Design:**

Observational descriptive study on the application and acceptance of the CTT. Retrospective case-control study for SARS-CoV-2 transmission risk factor determination and for evaluation of the CTT's risk stratification algorithm. **Setting**: Tertiary hospital in Germany.

**Participants:**

3514 contacts of hospital staff members to 322 SARS-CoV-2-positive cases.

**Methods:**

A case-control study was performed to identify risk factors for SARS-CoV-2 transmission and for unprotected contacts among staff members. To evaluate strengths and weaknesses of the CTT performance statistics were analyzed and users completed a questionnaire measuring satisfaction and acceptance of the tool.

**Results:**

In 2021, the CTT was used for the algorithm-based semi-automated management of 3514 in-hospital contacts. The tool determined the risk category of individual contacts and generated messages for the information of the local public health department, the in-hospital SARS-CoV-2 test center and all staff members who had contact to the index case. Staff members without regular contacts to patients had significantly (*P*<0.005) more unprotected contacts to other staff members (25.5% vs. 9.6%) and more SARS-CoV-2 transmissions per contact (4.9% vs. 0.6%) than staff members with frequent contacts to patients. The profession “nurse or medical technical service” was associated with significantly (*P*<0.005) more unprotected contacts between staff members (11.0% vs. 2.6%) compared to the profession “physician”.

**Conclusions:**

Digital tools can increase the efficiency of in-hospital contact tracing. The CTT enable a timely systematic analysis of risk factors among staff members.

## Background

In late 2019, the outbreak of a novel viral pneumonia, which later became known globally as COVID-19, occurred in the city of Wuhan, China. Common symptoms of the disease include fever, cough, chest discomfort, olfactory and gustatory disturbances and in severe cases, dyspnea and bilateral lung infiltration. [[1], [2], [3]] SARS-CoV-2 is transmitted by exposure to virus-containing droplets and aerosols. Mask-wearing requirements are effective in controlling the pandemic, by reducing transmission of these virus-containing respiratory particles. [4] Vaccination against SARS-CoV-2 can provide partial protection against infection with the virus, whereby the protective effect depends on the vaccine, the number of vaccination doses, the time of the last vaccination dose and the virus variant of SARS-CoV-2. [[5], [6], [7]]

Contact tracing is one of the most effective containment measures for SARS-CoV-2. [8] The aim of contact tracing is to identify and quarantine potentially infected contacts before the onset of symptoms and thus to prevent onward transmission. [9] This is particularly important since some SARS-CoV-2 infected persons may not exhibit signs or symptoms of illness at all. [10] Structured contact tracing is also important to reduce staff absences in hospitals and to avoid nosocomial outbreaks among patients, who often have risk factors for severe COVID-19. [11] In Germany, the management of SARS-CoV-2 contacts was largely specified by the Robert Koch Institute as national public health authority. [12] Particularly when dealing with contacts among medical staff, infection protection on one hand and the maintenance of acute medical care on the other hand competed with each other. [13].

With growing numbers of SARS-CoV-2-positive staff members, in November 2020 our paper-based procedure for in-hospital contact tracing reached its limits due to the high time requirements per case. For this reason, a spreadsheet-based contact tracing tool (CTT) was developed to automate essential steps of the hospital's contact tracing process. Here we report on the lessons learned from one year of semi-automated contact tracing in our hospital.

## Methods

### Setting

The University Hospital Magdeburg is a tertiary hospital located in Central Germany. It has (state August 2022) 4706 staff members and treats about 50,000 patients annually. During the whole pandemic, the Section Infection Control (SIC) performed COVID-19 contact tracing among hospital's staff and inpatients. The SIC consists of two infection control physicians and seven infection control nurses.

### Description of the contact-tracing-tool

**Figure 1:**
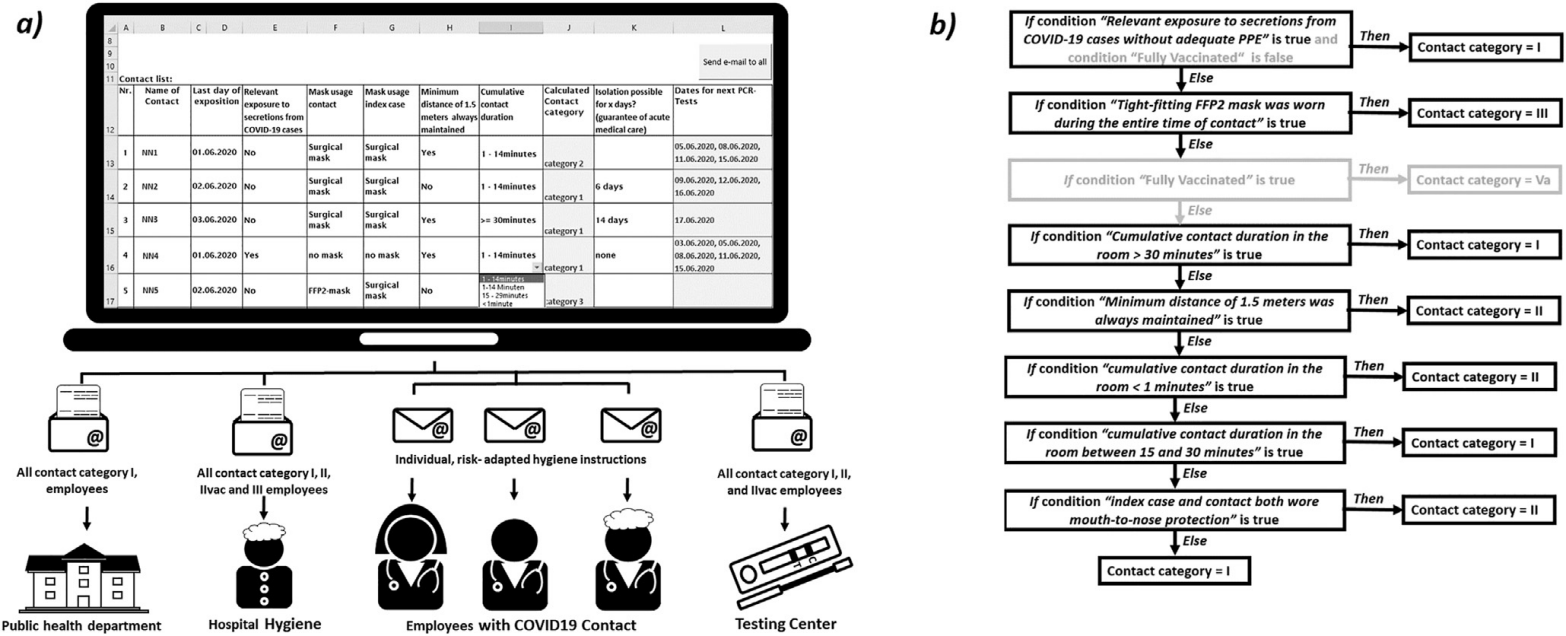
a) Workflow of the CTT. The spreadsheet is mailed to the index case or a supervisor. After filling the spreadsheet data, the risk category for contacts is assigned according to the algorithm shown in figure b and E-mail messages are send to the in-hospital testing center to make appointments for PCR testing, the public health department in order to obtain quarantine certificates, the in house infection control department of the hospital, and to all contacts in order to instruct staff members about the individual contact precautions that are necessary according to their individual risk categories. b) Algorithm for determining the risk category for contacts between hospital staff and a SARS-CoV-2-positive person based on various risk factors. Due to changes in the regulatory framework, the algorithm was slightly modified in June 2021 (changes shown in grey).

The CTT was developed in cooperation between the SIC and the hospital's IT department using Microsoft Excel 2016 and Visual Basic for Applications. Contacts were traced from 48 hours before symptom onset. The core of the CTT was a spreadsheet containing the contact list (Figure 1a). For each contact, name, date of the last exposition, exposure to secretions, use of medical masks during the contact - for each the contact and the index case, consistent distance maintenance of 1.5 meters and cumulative duration of the contact had to be recorded. In July 2021, a query about the vaccination status of the contacts was added due to adjustments in the German Quarantine Ordinance. Based on this information, the spreadsheet calculated the risk category using the algorithm shown in Figure 1b that was based on the recommendations of the Robert Koch Institute. For each health care worker with category 1 contact, the public health department and the management level of the hospital determined individually whether 14 days of quarantine could be observed, or whether the quarantine period had to be shortened to 6 days or even had to be omitted under repetitive PCR-testing in order to maintain acute medical care. The dates for PCR testing were calculated by the CTT according to the last date of exposure and the determined risk category.

Mailing automation was realized by a macro using the Visual Basic's Outlook Application object and the Workbook. ExportAsFixedFormat function (for PDF creation). In the manner just described, lists with either category 1 contacts only, category 1, 2 and 2 vac contacts or all contacts were send to the public health department, to the in-hospital SARS-CoV-2 testing center, and to the SIC, respectively. The CTT also sent individual emails to all staff members on the contact list that informed about the requirement and the dates of PCR-testing and about the necessary contact precautions according to the contact's calculated risk classification.

The spreadsheet was mailed to the SARS-CoV-2 positive employee and/or the supervisor. The spreadsheet data then were filled either by the quarantined employee according to his/her memories of contacts or on the supervisor's workplace computer after directly calling the index case and/or interviewing the coworkers about their contacts to the index case. Inquiries to the infection control team regarding the operation of the tool occurred but were uncommon (less than 25% of cases) and could usually be resolved within a few minutes. The data supplied by health care workers on the list were not double-checked by the SIC.

### Evaluation of the CTT among infection control staff

In order to evaluate strengths and weaknesses of the CTT, the infection control nurses of the hospital (n = 7), who worked with the CTT, completed a questionnaire, which was composed of 16 items measuring the acceptance of various sub-aspects of this form of semi-automated contact tracing (Table II). The items were rated on a 5-point Likert scale, ranging from zero (strongly disagree) to four (strongly agree).

**Table I:** Contacts traced with the CTT from 01.01.2021 to 31.12.2021

Number of …	
index cases	322
PCR test appointments that were arranged automatically	1719
Number of staff members …	
for whom the algorithm-based risk assessment was performed	3514
who were classified as contact category I	151
and had 14 days of quarantine	80
and had 6 days of quarantine	33
and had no quarantine (maintenance of acute medical care)	38
who were classified as contact category II	161
who were classified as contact category IIvac	353
who were classified as contact category III	2849
who got a risk-adapted hygiene instructions via email for employer	3514
who have been automatically reported to the health department	151
who have been automatically reported to the hospital hygiene	3514
who have been automatically reported to the testing center	665

**Table II:** Results of the questionnaire that was completed by the in-hospital infection control nurses (n=7) who used the CTT tool from 01.01.2021 to 31.12.2021

Statement	0 strongly disagree	1 disagree	2 undecided	3 Agree	4 strongly agree
Overall, I prefer an electronic list (Excel via email) to a paper solution (form and fax).	0%	0%	0%	0%	100%
Compared to the paper solution, the electronic form simplifies things for me:					
a) sending (email vs. fax)	0%	0%	0%	0%	100%
b) readability	0%	0%	0%	14%	86%
c) archiving	0%	0%	0%	0%	100%
I prefer the classification of risk groups on the basis of a fixed algorithm to an individual classification.	0%	0%	0%	14%	86%
The automated reporting of Cat. 1 staff members to the health department has relieved the work process.	0%	0%	14%	0%	86%
The automated reporting of Cat 1 and Cat 2 staff members to the test center has relieved the work process.	0%	0%	0%	29%	71%
The automated calculation of the test days has relieved the work process.	0%	0%	0%	0%	100%
The automated creation of emails with individual contact precaution measures according to risk was helpful.	0%	0%	0%	0%	100%
The physicians were aware of the content of the personal emails	0%	14%	43%	14%	29%
The nurses were aware of the content of the personal emails	0%	14%	57%	14%	14%
I often had technical problems with list	0%	71%	14%	0%	14%
The health care workers often had technical problems with list	0%	43%	43%	14%	0%

### Performance analyses and statistics

All contact lists sent to SIC were analyzed retrospectively. The number of category 1, 2, 2 vac and 3 contacts were counted, as well as the number of PCR-appointments and emails send to the staff members, the public health department and the testing center. In order to determine the SARS-CoV-2 transmission rates, contacts were compared with the list of positive staff members. Transmission was assumed if infection occurred within 14 days of contact and no alternative source of infection (e.g. positive family member) was considered more likely. The data on gender, workplace and profession were added for the index cases. Data analysis were performed using Microsoft Excel 2016. The chi square test was used for the assessment of significant associations between risk factors and outcome variable with a significance level of *P* < 0.005 in order to correct for multiple comparisons. Chi square test was also used to examine whether the risk classes determined by the algorithm allow significant predictions about the expected infection risks.

## Results

From January 1^st^ to December 31^th^ 2021, SARS-CoV-2 was detected by PCR in 334 staff members, which corresponds to a seven-day incidence of 136 cases per 100.000. Further, the virus was detected by PCR in 182 in- or outpatients that were not treated primarily because of COVID-19 (incidental finding at admission screening or symptom onset during the hospital stay). Contact tracing was performed for 322 SARS-CoV-2-positive index cases (patients or staff members) using the CTT (Table I). The discrepancy between the total number of cases (516 patients and staff members) and the number of cases followed up by CTT (n=322) is due to the fact that some staff members had no contact to the hospital during their infectious period (e.g. due to holidays/they already were in quarantine) or because within a SARS-CoV-2 outbreak other infection control measures were implemented (e.g. screening all patients and staff members of the ward every second day). Medical staff that used adequate PPE in settings with previously known COVID-19 patients were generally not considered as contact. Contacts of patients with SARS-CoV-2-positive cases were not traced using the CTT.

Despite mandatory in-hospital contact restrictions, the 322 index cases together had 3514 contacts to hospital staff members (maximum: 47; minimum: 1; mean: 10.9; median: 9). Of the 3514 contacts, 151 (4.3%) were classified as category 1 contacts (mainly face to face contact or stay in the same room as the index for more than 30 minutes), 161 (4.6%) as category 2 contacts (no face to face contact but stay in same room as the index for less than 30 minutes), 353 (10.1%) as category 2 vac contacts (since June 2021; face to face contact or stay in the same room as the index without FFP2-mask but contact is fully vaccinated) and 2849 (81.8%) as category 3 contacts (use of a FFP2 mask during the whole contact).

All staff members with category 1 contacts were reported automatically to the public health department by the CTT. The public health department ordered a quarantine of 14 days for 80 of these 151 staff members. For 33 staff members the quarantine was shortened to 6 days and no quarantine was ordered for 38 staff members in order to maintain acute medical care. In addition, the CTT automatically arranged 1719 appointments for PCR testing at the hospital's testing centre for the 655 staff members with contacts of category 1, 2 or 2 vac. Regardless of the risk classification, all 3514 contacts were reported to SIC by the CTT. Finally, the CTT automatically generated and sent 3514 emails, one for each contact, that informed about the necessary infection control measures. Staff members with private SARS-CoV-2 contacts (especially contact to positive family members) were not followed up using CTT. Among 289 private contacts, there were 55 transmissions, corresponding to a transmission rate of 19%.

Forty-nine staff members that were traced by the CTT became SARS-CoV-2-positive, which corresponds to a rate of 0.15 secondary infections per index case. The risk of SARS-CoV-2 transmission was dependent on the risk class of the contact (category 1: 11.3% (17/151); category 2: 3.1% (5/161); category 2 vac: 3.7% (13/353); category 3: 0.5% (14/2835). Thus it can be stated that the SARS-CoV-2 transmission risk differs considerably in the individual risk classes determined by the algorithm (Table III, which also includes the corresponding significance levels between the individual risk groups). The 49 secondary cases were generated by contact to 26 index cases.

**Table III:** Validation of risk groups

Risk category	Trans-missions	No	Total contacts	Rate	*P*-value compared to risk category		
		Trans.			Category 2	Category 2v	Category 3
Category 1	17	134	151	11,3%	<0,005^a^	<0.005^a^	<0.005^a^
Category 2	5	156	161	3,11%	-	0,2	<0,005^a^
Category 2v	13	340	353	3,68%	-	-	<0,005^a^
Category 3	14	2835	2849	0,49%	-	-	-

a asterisks indicate significance of the indicated *P*-value.

The analysis of contacts further showed that staff members without direct patient contacts had significantly (*P* < 0.005) more unprotected contacts among each other compared to staff members with regular close patient contacts (contacts classified category 1 or 2: 25.5% vs. 9.6%, Table IV). As a result, there were significantly (*P* < 0.005) more SARS-CoV-2 transmissions per contact in staff members without regular patient contacts compared to staff members with regular patient contacts (transmission risk over all contacts: 4.9% vs. 0.6%). Within the group of staff members with regular patient contacts, the physicians had significant (*P*<0.005) less unprotected contacts compared to the nursing staff/medical technical assistants (contacts classified category 1 or 2: 2.6% vs. 11%). The risk of SARS-CoV-2 transmission, however, was not significantly different among both groups. Gender (*P*=0.01) was no independent risk factor for a higher rate of unprotected contacts or SARS-CoV-2 transmission.

**Table IV:** Risk factors of the index case for higher risks contacts and for SARS-CoV-2 transmission

Risk factor	Cases	Controls	Total	Rate	*P*-value
**Staff members with and without regular patient contacts**					
*Higher risk contacts (cases = category 1/2 contacts; controls = category 2 vac/3 contacts)*					
regular patient contacts	139	1307	1446	9.6%	<0,005^a^
no regular patient contacts	166	486	652	25.5%	
*SARS-CoV-2 transmission (cases = transmission; controls = no transmission)*					
regular patient contacts	9	1437	1446	0.6%	<0,005^a^
no regular patient contacts	32	620	652	4.9%	
**Gender of index in workplaces close to the patient**					
*Higher risk contacts (cases = category 1/2 contacts; controls = category 2 vac/3 contacts)*					
Male	27	388	415	6.5%	0.01
Female	112	919	1031	10.9%	
*SARS-CoV-2 transmission (cases = transmission; controls = no transmission)*					
Male	1	414	415	0.2%	0.24
Female	8	1023	1031	0.8%	
**Profession of index case in workplaces with frequent contacts to patients**					
*Higher risk contacts (cases = category 1/2 contacts; controls = category 2 vac/3 contacts)*					
Physician	6	226	232	2.6%	<0,005^a^
other HCW	133	1081	1214	11.0%	
*SARS-CoV-2 transmission (cases = transmission; controls = no transmission)*					
Physician	0	232	232	0.0%	0.19
other HCW	9	1205	1214	0.7%	
**Patient vs. staff member as index**					
*Higher risk contacts (cases = category 1/2 contacts; controls = category 2 vac/3 contacts)*					
patient as index	7	1409	1416	0.5%	<0,005^a^
staff member as index	305	1793	2098	14.5%	
*SARS-CoV-2 transmission (cases = transmission; controls = no transmission)*					
patient as index	8	1408	1416	0.6%	<0,005^a^
staff member as index	41	2057	2098	2.0%	

a asterisks indicate significance of the indicated *P*-value.

Finally, staff members were significantly (*P*<0.005) more likely infected after contact to SARS-CoV-2-positive staff members than after contact to SARS-CoV-2-positive patients. Among the 49 SARS-CoV-2 transmissions to staff members, other staff members were the source of infection in 41 cases (secondary attack rate (SAR) 2.0%) and patients in eight cases (SAR 0.6%). In 2021, 131 SARS-CoV-2-positive in- or outpatients had 1416 contacts to hospital staff members before they were diagnosed SARS-CoV-2-positive, either as an incidental finding at the routine PCR-test at admission or during the in-hospital stay. As FFP2 filtering facepiece respirators were compulsory for staff members to use for all patient contacts throughout 2021, 99% of contacts were category 3 contacts and less than 1% were category 2 or 2vacc contacts.

In order to evaluate the CTT, all infection control nurses of the hospital (n=7) received a questionnaire. The results are shown in Table II. Despite some users reporting minor technical issues, the CTT was clearly preferred over the previously used paper-based solution due to time savings by algorithm-based risk class calculation and automated mailing processes. We have no quantitative data on the amount of time spent for contact tracing among members of the infection control team. In November 2020 the infection control nurses were at their absolute limit with the contact-tracing of 33 index cases using a paper-based procedure. After implementation of the CTT in January, November, December 2021 between 70 and 80 cases and in February 2022 even 116 index cases were followed up without major problems. Thus, using the CTT allowed time savings up to 75%.

## Discussion

At the very beginning of the SARS-CoV-2 pandemic, health-care workers faced an elevated risk of unprotected exposure to the virus. [14] In contrast, after implementation of universal masking in the later phase of the pandemic healthcare workers had a similar or even lower SARS-CoV-2 incidence compared to the general population of the same geographical despite exposure to SARS-CoV-2-positive patients. [15,16] In our study the seven-day-SARS-CoV-2 incidence among our staff members in 2021 was 11% below the incidence of the general population in Saxony-Anhalt (136 vs. 152 per 100.000).

From 334 SARS-CoV-2-positive staff members, 49 have been likely infected in the working environment, while 272 infections were considered as not hospital-acquired. In 13 cases, a link to a SARS-CoV-2 outbreak seems possible even if no plausible transmission route could be identified. The SAR of our staff members in cases of contacts to SARS-CoV-2-positive family members was 19%, while the rate for hospital contacts was 1.5% across all risk classes. In a meta-analysis, Thomson *et al.* gave a pooled SAR of 21.1% [95% CI 17.4–24.8] for household contacts and a pooled SAR for hospitals of 3.4% [95% CI 1.0–6.9]. [17] Our SAR within the hospital is thus in the lower range of this confidence interval.

Staff members were significantly more likely to be infected by other SARS-CoV-2-positive staff members than by SARS-CoV-2-positive patients, as described by Emecenet *et al.* and Schneider *et al.* [18,19].

Despite strict in hospital contact restrictions, SARS-CoV-2-positive index cases had an average of 10.9 contacts. Despite identical contact precaution rules, staff members without regular patient contacts (e.g.: laboratory staff, administrative staff, technicians) had significantly more unprotected contacts among each other than staff members with regular close patient contacts (e.g. physicians, nurses), which paradoxically resulted in a significant higher risk of virus transmissions among staff members with overall less person to person contacts. This reinforces previous findings [20,21] that exposure at the workplace was only a minor risk factor for HCW compared to community risk factors after universal masking was implemented. We speculate that the higher compliance of staff working close to patients can be explained in particular by their sense of responsibility towards their vulnerable patients.

Within the group of staff members working close to the patient, in 2021 physicians had significantly fewer unprotected contacts compared to nursing/medical technical staff. We have no data on the total number of contacts of nurses or physicians to patients or co-workers, respectively, which limits the interpretation of the data and thus we cannot draw conclusions in regard to the infection control compliance among both professional groups. In contrast to nursing/medical technical staff, physicians often have an own office room, so that it is easier to spend meal and rest breaks alone than if there is only one shared recreation room on the ward for the whole team.

In our study 11.3 % of staff members with category 1 contacts became SARS-COV-2-positive within 14 days. This value essentially corresponds to the transmission values reported in the literature for unprotected contacts in hospitals (Bailie *et al.*: 11% [22]; Lupia *et al.* 11.7% for confirmed cases only (13.1% for confirmed, probable and suspected cases) [23]; Zirbes *et al.* 11.3% [24]). Conversely, however, this means that 89% of the staff in this group could not continue to work for 14 days, even though they did not subsequently acquire a SARS-CoV-2 infection. In consultation with the health department, 71 of the 151 Category 1 contacts were able to shorten or suspend quarantine with PCR testing every second day and continuous wearing of an FFP2 mask in order to maintain acute medical care. To the best of our knowledge, there were no infections originating from these staff members.

In response to the COVID-19 pandemic, different digital tools have been developed to assist contact tracing [25]. The software used for in-hospital contact tracing and the sub-steps for which these tools enable automation differ considerably. So Bailie *et al.* [22] performed hospital-based contact-tracing using REDCap. Nevertheless, communication between SARS-CoV-2 contacts and the contact tracing team took place via telephone interview. Zirbes *et al.* [24] describe the development of a web-based contact tracing and point-of-care-testing workflow. Lupia *et al.* [23] first used REDCap and Go. Data software, a tool developed by WHO, for early outbreak investigation. From summer 2021, however Go. Data was replaced by an in-house solution, because it required multiple manual steps and did not allow automation of the follow-ups. Other studies describe the use of clinical data from the electronic health record or Bluetooth devices to augment healthcare worker contact tracing during the COVID-19 pandemic [26, 27].

## Conclusions

In summary, digital tools can increase the efficiency of in-hospital contact tracing. Despite some users reporting minor technical issues, the CTT proved to be an acceptable local solution for contact tracing in our hospital. By avoiding time consuming personal interviews, it reduced the time requirements for contact tracing, while simultaneously improving the data quality by avoiding an individual classification of risk-groups and enabling timely analysis of the compiled data. In principle, after adapting the parameters of the algorithm to the corresponding properties of a pathogen (e.g. incubation time, prevention by protective equipment) this type of contact tracing can be used to trace other pathogens. In our opinion, the benefit of the tool is highest for infections with relatively long incubation periods, high reproductive numbers, and pre-symptomatic pathogen shedding or oligo- or asymptomatic courses. Also, others described local solutions that were born out of necessity at the beginning of the pandemic and were adapted later (1) (2). In-hospital epidemiological data are often available in situations, when general testing outside the hospital is not yet available e.g. because of limited diagnostic resources. By displaying the data in real time, e.g. in the form of a dashboard, in-hospital infection control teams as well as regional infection control authorities could quickly react on new trends in the spread of the pathogen.

## CRediT author statement

Lukas Bechmann MD: Conceptualization; Writing - Original Draft; Methodology; Data Curation; Investigation; Software; Visualization.

Gernot Geginat MD: Conceptualization; Writing - Review & Editing; Visualization; Methodology; Validation; Supervision.
